# Supplementary material for: Dysregulation of Muscle-Specific MicroRNAs as Common Pathogenic Feature Associated with Muscle Atrophy in ALS, SMA and SBMA: Evidence from Animal Models and Human Patients
Source: Int J Mol Sci. 2021 May 26;22(11):5673. doi: 10.3390/ijms22115673 (PMC8198536; doi:10.3390/ijms22115673)
Supplement: Supplementary file 1 [file ijms-22-05673-s001.zip › ijms-1210198-supplementary.pdf]

Supplementary Materials

Denervation mouse model

Three month-old male mice (C57BL/6 strain, $n = 5$) were anaesthetized with ketamine and xylazine (respectively 90 mg/kg IP and 12.5 mg/kg IP). An incision was made through the skin and the upper region of the left gluteal muscle to expose the sciatic nerve, which was then cut 1–2 mm distal to the sciatic notch. The proximal portion of the nerve was sutured to prevent errant reinnervation of the gastrocnemius muscle. Right sciatic nerve was only exposed and utilized as sham internal control in each animal. C57BL/6 strain ($n = 5$) not subjected to denervation was included as control. Mice were sacrificed 7 and 21 days later, and gastrocnemius muscles were collected, snap frozen and conserved at -80 °C.

Supplementary Figures and Tables

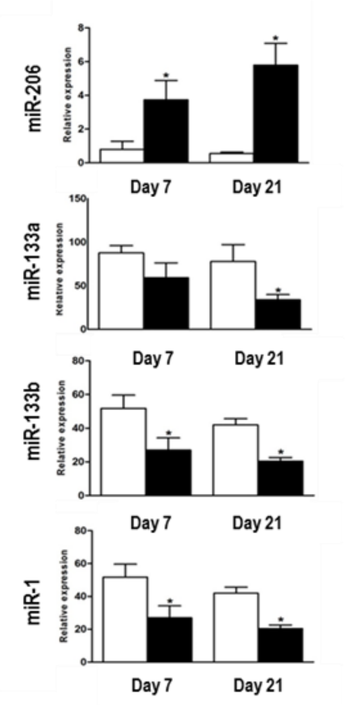

Figure S1. Expression levels of myomiRs in muscle tissue of denervated and control mice at day 7 and 21. Quantitative real-time PCR expression analysis of miR-206, miR-133a, miR-133b and miR-1 in muscle tissue of control (white bars) and denervation (black bars) mouse model at day 7 and 21. Expression levels are reported as mean ± SEM of $2^{-\Delta Ct}$ values normalized against the endogenous control U6 ($n = 5$ animals for each group). *$p < 0.05$, Mann-Whitney test.

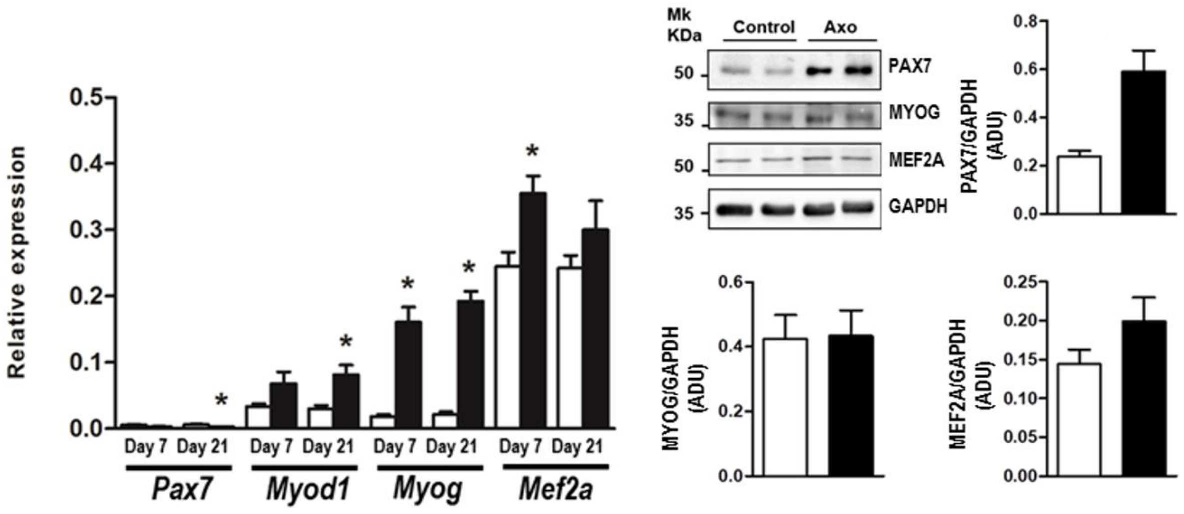

Figure S2. Altered expression of the predicted myomiR target genes and their encoding proteins in muscle tissue of denervated and control mice. Left graph, quantitative real-time PCR expression analysis of *Pax7*, *Myod1*, *Myog* and *Mef2a* genes in gastrocnemius tissue from control (white bars) and denervation (black bars) mouse model (n = 4 animals per group). For each gene, differences between denervated and control animals were found and detected at the different stages of denervation (day 7 and 21). Thus, in the graph we have reported the mean relative gene expression levels ± SEM of each gene calculated in each animal group at day 7 and 21 (n = 8). Expression levels are reported as mean ± SEM of $2^{-\Delta Ct}$ values normalized against the endogenous control *Rplp0*. *p < 0.05, **p < 0.01, ***p < 0,001, Mann-Whitney test. Right panel, representative Western blot analysis of PAX7, MYOG and MEF2A protein, with relative densitometric analysis, in gastrocnemius tissue of denervated (black bars) and control (white bars) animals at day 21 (n = 3 animals per group). No statistical significance was observed. Density values are reported as mean ± SEM, corrected for background and normalized to the GAPDH control; Mann-Whitney test.

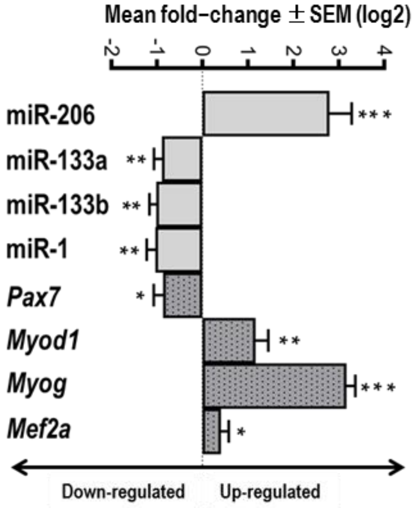

Figure S3. Altered expression of myomRs and their predicted target genes in muscle tissue of denervation model. To represent the expression of myomiRs and their predicted targets, we first calculated the $2^{-\Delta Ct}$, by normalizing myomiR and target expression for the denervation model and at each time point towards U6 and 18S respectively; the fold change was estimated by dividing the $2^{-\Delta Ct}$ of pathological mice versus $2^{-\Delta Ct}$ control values. Then the log2 of fold change was shown in Figure. Data are presented as mean ± SEM of log2 of fold changes of $2^{-\Delta Ct}$ expression of myomiRs (light grey bars) and mRNA targets (grey with dots bars), obtained at day 7 and 21 relative to control mice. *p < 0.05, **p < 0.01, ***p < 0.001, Mann-Whitney test.

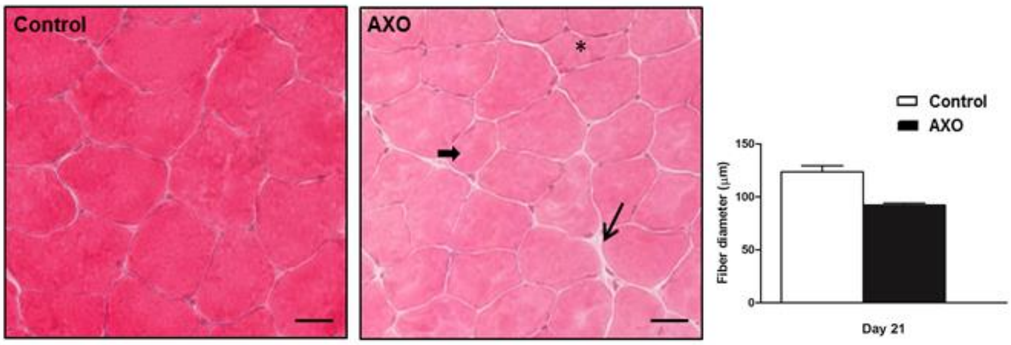

Figure S4. Histological analysis of gastrocnemius muscle in denervated and control mice. Representative transversal hind limb muscle sections, stained with hematoxylin/eosin, in denervated mice (right column) at day 21 after sciatic nerve cut, and relative age-matched controls (left column). Long arrows indicate slightly enlarged endomysial spaces; asterisks indicate angulated muscle fibers; short arrows indicate hypotrophic fibers. Magnification 40×. Scale bar = 10 μm. Left panel, measurement of fiber diameter in muscle of denervated mice at day 21 after sciatic nerve cut (black bar), and relative age-matched control mice (white bar). Each histogram represents the mean diameter (μm) ± SEM of muscle fibers measured in four muscle sections per mouse from three mice in each group. Mann-Whitney test.

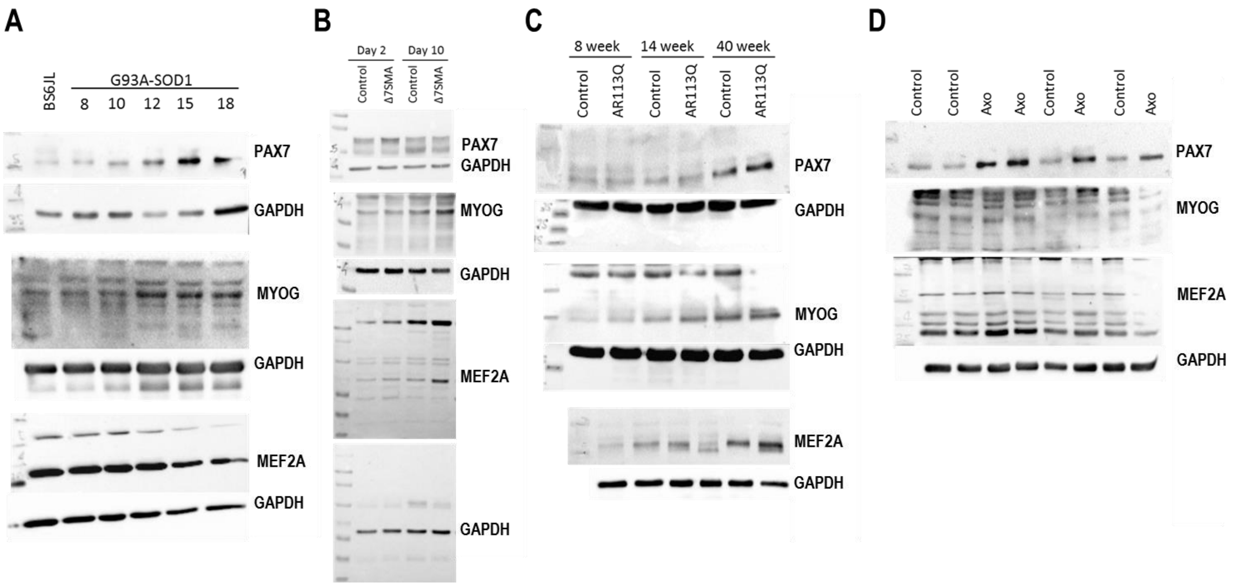

Figure S5. Original images of western blots reported in Figure 3 and Supplemental Figure 2. Representative Western blot analysis of PAX7, MYOG and MEF2A proteins in in muscle tissue of: (**A**) G93A-SOD1 mice at week 8, 10, 12, 15, 18 and B6.SJL mice at 8 weeks; (**B**) Δ7SMA and control mice at day 2 and 10; (**C**) AR113Q and control mice at week 8, 14, 40; (**D**) denervated and control mice. Western blot images of PAX7, MYOG, MEF2A and GAPDH proteins derived from the same electrophoretic gels. Western blot protocol was optimized by cutting the membrane into small stripes at proper molecular weight, that were incubated separately with anti-PAX7, anti-MYOG, anti-MEF2A and anti-GAPDH antibodies, a "stripe puzzles" method reported in literature [Colciaghi, F. et al. Targeting PSD95-nNOS interaction by Tat-N-dimer peptide during status epilepticus is neuroprotective in MAM-pilocarpine rat model. Neuropharmacology 2019, *153*, 82–97.

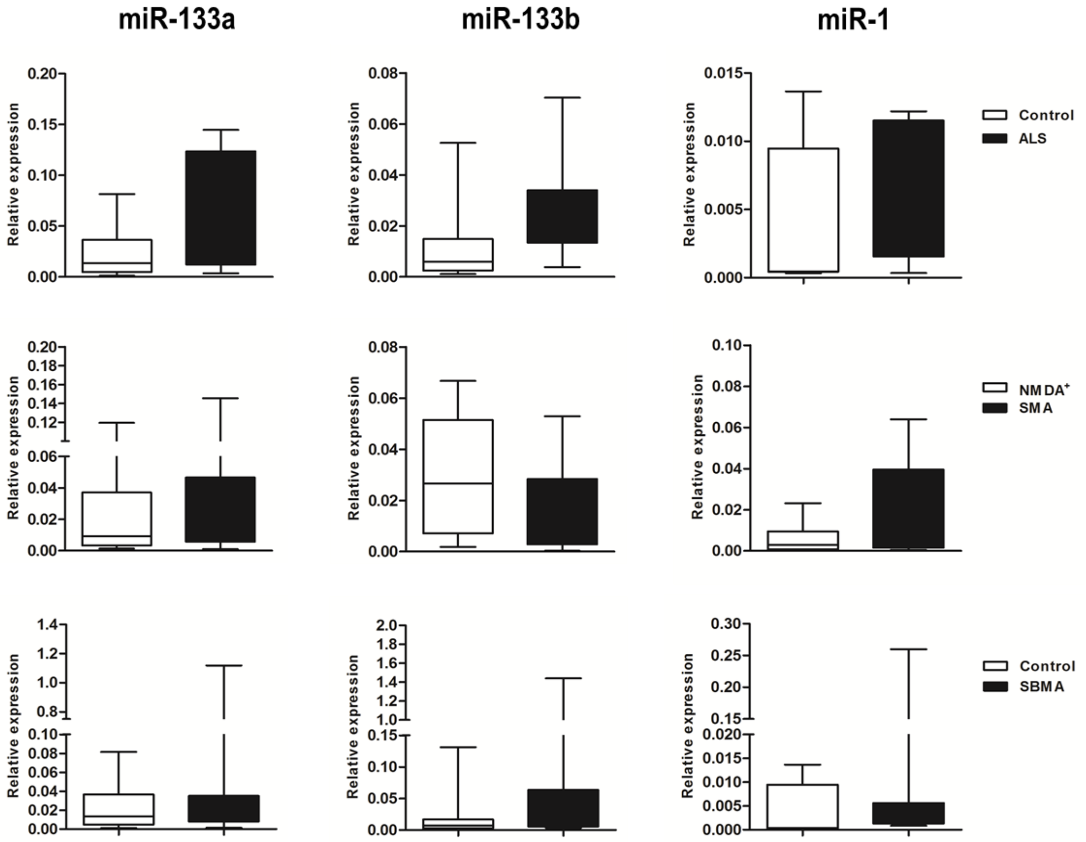

Figure S6. Quantitative real-time PCR analysis of myomiRs, miR-133a, miR-133b and miR-1, in serum of ALS, pediatric SMA and SBMA patients. Quantitative real-time PCR analysis of myomiRs in total RNA extracted from serum patients (black bars), healthy controls (white bars) and patients with anti-NMDA receptor encephalitis (white bars in the SMA graph) as controls for pediatric SMA. Relative expression data are presented as mean ± SEM of $2^{-\Delta Ct}$ values normalized against the endogenous control miR-16. Mann-Whitney test.

Table S1. List of primer sequences of the selected myomiR target genes. Sequences of primers used in quantitative real-time PCR reactions to assess the expression of the putative target genes of myomiRs, *Pax7*, *Myod1*, *Myog* and *Mef2a*.

Rplp0	**5′-GGT GCC ACA CTC CAT CAT CA-3′ (forward)**
	5′-AGG CCT TGA CCT TTT CAG TAA GT-3′ (reverse)
Pax7	5′-GTA TGG CCA AAC TGC TGT TGA T-3′ (forward)
	5′-GGA GTG TTC CCC AAG CTT CA-3′ (reverse)
Myod1	5′- GGC TAC GAC ACC GCC TAC TA-3′ (forward)
	5′-GTG GAG ATG CGC TCC ACT AT-3′ (reverse)
Myog	5′-GGG CAA TGC ACT GGA GTT-3′ (forward)
	5′-CAC GAT GGA CGT AAG GGA GT-3′ (reverse)
Mef2a	5′-CAG CCA GCT CAA CAT TAG CA-3′ (forward)
	5′-GGC ACC GTG AGG TCT GTA GT-3′ (reverse)

Table S2. Results of Spearman myomiR/mRNA target correlation in G93A-SOD1, Δ7SMA and AR113Q mouse muscle. Correlation analyses to assess positive or negative correlation between the expression levels of each myomiR and the mRNA levels of the target genes in G93A-SOD1, Δ7SMA and AR113Q mice at the different disease stages.

myomiRs	Target Genes	G93A-SOD1	Δ7SMA	AR113Q
**miR-206**	*Pax7*	**r_s= -0.613; *p*= 0.006**	$r_s < +0.5$	$r_s < +0.5$
	Myod1	**r_s = 0.587; *p*= 0.010**	$r_s < +0.5$	$r_s < +0.5$
	Myog	$r_s < +0.5$	$r_s < +0.5$	$r_s < +0.5$
	Mef2a	$r_s < +0.5$	$r_s < +0.5$	$r_s < +0.5$
**miR-133a**	*Pax7*	$r_s < +0.5$	**r_s = −0.672; *p* = 0.039**	$r_s < +0.5$
	Myod1	$r_s < +0.5$	$r_s < +0.5$	**r_s = 0.628; *p* = 0.012**
	Myog	$r_s < +0.5$	$r_s < +0.5$	$r_s < +0.5$
	Mef2a	$r_s > -0.5$	$r_s > +0.5$	$rs < +0.5$
**miR-133b**	*Pax7*	$r_s < +0.5$	$r_s < +0.5$	$r_s < +0.5$
	Myod1	$r_s < +0.5$	$r_s < +0.5$	**r_s = 0.803; *p* = 0.0003**
	Myog	$r_s < +0.5$	$r_s < +0.5$	**r_s = 0.775; *p* = 0.0007**
	Mef2a	$r_s < +0.5$	$r_s < +0.5$	$r_s < +0.5$
**miR-1**	*Pax7*	$r_s < +0.5$	**r_s = −0.685; *p*= 0.035**	**r_s = 0.620; *p* = 0.023**
	Myod1	$r_s < +0.5$	$r_s < +0.5$	**r_s = 0.573; *p* = 0.002**
	Myog	$r_s < +0.5$	$r_s < +0.5$	**r_s = 0.714; *p* = 0.006**
	Mef2a	$r_s < +0.5$	**r_s = 0.676; *p* = 0.031**	$r_s < +0.5$
